# Intake of Vitamin and Mineral Supplements and Longitudinal Association with HbA_1c_ Levels in the General Non-Diabetic Population—Results from the MONICA/KORA S3/F3 Study

**DOI:** 10.1371/journal.pone.0139244

**Published:** 2015-10-16

**Authors:** Sigrid Schwab, Astrid Zierer, Margit Heier, Beate Fischer, Cornelia Huth, Jens Baumert, Christa Meisinger, Annette Peters, Barbara Thorand

**Affiliations:** 1 Institute of Epidemiology II, Helmholtz Zentrum München, German Research Center for Environmental Health, Neuherberg, Germany; 2 Department of Epidemiology and Preventive Medicine, University of Regensburg, Regensburg, Germany; 3 German Center for Diabetes Research (DZD e.V.), Neuherberg, Germany; 4 MONICA/KORA Myocardial Infarction Registry, Central Hospital of Augsburg, Augsburg, Germany; Sookmyung Women's University, REPUBLIC OF KOREA

## Abstract

**Background:**

Lower levels of hemoglobin A_1c_ (HbA_1c_) are associated with a decreased risk of cardiovascular complications in diabetic and non-diabetic individuals. The aim of the study was to longitudinally investigate the association between the use of 11 vitamins and minerals (vitamins E, C, D, B_1_, folic acid, carotenoids, calcium, magnesium, zinc, iron, and selenium) and change in HbA_1c_ levels over 10 years in non-diabetic individuals drawn from the general population.

**Methods:**

Baseline data were available from 4447 subjects included in the population-based “Monitoring of Trends and Determinants in Cardiovascular Diseases” (MONICA) Augsburg S3 survey (1994/95). Follow-up data were derived from 2774 participants in the follow-up survey named “Cooperative Health Research in the Region of Augsburg” (KORA) F3 (2004/05). Vitamin/mineral intake from supplements and medications was assessed in a personal interview, where participants were asked to bring product packages of preparations that had been ingested during the last 7 days prior to the examination. Associations between regular vitamin/mineral intake amounts and HbA_1c_ levels measured at baseline and follow-up were investigated using generalized estimating equation models. For carotenoids, analyses were stratified by smoking status.

**Results:**

None of the investigated nutrients except for carotenoids was significantly associated with changes in HbA_1c_ levels after 10 years. Regular intake of carotenoids from supplements and medications in amounts > 6.8mg/d (upper tertile) was associated with an absolute –0.26% (95% CI: –0.43 to –0.08) lower increase in HbA_1c_ levels compared with no intake of carotenoids. An inverse association was observed in those who never smoked but not in (former) smokers.

**Conclusion:**

Larger prospective and intervention studies in non-diabetic/non-smoking individuals are needed to confirm the results and to assess whether the observed associations between carotenoid intake and change in HbA_1c_ levels are causal. If our results are confirmed, high carotenoid intake could be one strategy for the prevention of cardiovascular complications in non-diabetic people.

## Introduction

Hemoglobin A_1c_ (HbA_1c_) reflects the percentage of glycated hemoglobin, which results from non-enzymatic attachment of glucose to hemoglobin. Levels of HbA_1c_ indicate the long-term average blood glucose level over the previous 8–12 weeks [1]. Values ≥ 6.5% can be used for the diagnosis of diabetes [2]. Besides its use as a diagnostic tool, HbA_1c_ has been investigated as a marker for cardiovascular risk. An increase in HbA_1c_ levels of 1% is associated with a 17% increase in risk of cardiovascular disease and a 15% increase in risk of all-cause mortality in subjects with type 2 diabetes [3]. The increased risk of cardiovascular complications with higher HbA_1c_ levels is not only evident in those with established diabetes, but also in non-diabetic adults [4–7]. In the latter, HbA_1c_ levels are more strongly associated with coronary heart disease risk than other glycemic measures [8], and all-cause mortality risk increases by 26% with a 1% increase in HbA_1c_ concentration [9]. Therefore, HbA_1c_ should be investigated continuously in the assessment of cardiovascular and mortality risk [9].

Previous studies on the association between vitamins/minerals and HbA_1c_ levels investigated the effect of vitamin E, vitamin C, vitamin D, vitamin B_1_, folic acid, carotenoids (β-carotene), calcium, magnesium, zinc, iron, and selenium [10–33]. For most nutrients, the results were not consistent between studies that investigated the same nutrients as exposure. Furthermore, all the above mentioned intervention studies were conducted in diabetic patients or high risk populations [10–27]. However, supplementation of nutrients might be less effective if the disease has already developed [11,14]. Therefore, investigations in non-diabetic populations are warranted. Population-based studies conducted to date have been of cross-sectional design [28,29,32,33], which precludes conclusions regarding causal relationships. Considering that chronic diseases develop over a period of several years, it is particularly important to conduct long-term studies to investigate potential prevention strategies.

To our knowledge, there is currently no population-based longitudinal study of the association between the use of different vitamin or mineral supplements and change in HbA_1c_ levels in the general healthy population. As the use of dietary supplements could be a promising and feasible strategy to prevent hyperglycemia related complications, the aim of the study was to investigate the longitudinal relationship between the intake of vitamins/minerals from supplements and medications with HbA_1c_ concentrations over 10 years in a population-based sample of non-diabetic people.

## Methods

### Study description and population

Baseline data were available from the population-based Monitoring of Trends and Determinants in Cardiovascular Diseases (MONICA) Augsburg S3 survey conducted in the years 1994/95 as part of the international World Health Organization (WHO) MONICA project [34]. The study region comprised the city of Augsburg (Germany) and its two surrounding counties. A total of 6640 participants aged 25–74 years were randomly selected by a two-stage cluster sampling method, of whom 4856 participated (73.1%) [35]. Of these, 3006 participants took part in the subsequent Cooperative Health Research in the Region of Augsburg (KORA) F3 survey after 10 years of follow-up [36]. After exclusion of diabetic individuals at baseline, either clinically diagnosed (n = 241) or with baseline HbA_1c_ levels ≥ 6.5% (n = 57), those who ingested antidiabetic medication at baseline or follow-up (n = 109), and participants with missing HbA_1c_ values at baseline and follow-up (n = 2), the final study population comprised 4447 subjects at baseline and 2774 at follow-up.

The study was conducted according to the guidelines laid down in the Declaration of Helsinki, and all procedures involving human subjects were approved by the ethics committee of the Bavarian Chamber of Physicians (Munich, Germany). Written informed consent was obtained from all participants.

### Data collection

#### Outcome

The outcome HbA_1c_ was assessed at baseline (1994/95) and at follow-up (2004/05). Non-fasting blood samples were collected according to a standardized procedure. HbA_1c_ was measured with turbidimetric immunoassays (Tina-quant® Hämoglobin A_1c_, Boehringer Mannheim, Mannheim, Germany, and Dimension Rxl 5 Dade-Behring (TINA) with coefficients of variation (CVs) < 5%) at baseline and follow-up. HbA_1c_ was treated as a continuous variable in all analyses.

#### Exposure

Intake of vitamins/minerals from supplements and medications was assessed in a personal interview at baseline (1994/95) and follow-up (2004/05) and also in between after 3 years from baseline investigation through a self-administered postal questionnaire in 2998 individuals (1997/98). Data from the postal questionnaire were only used for descriptive purposes. In the baseline and follow-up surveys, participants were asked to bring all product packages of preparations that were ingested during the last 7 days prior to the examination to the study center. In MONICA S3 (1994/95), the information was recorded through a questionnaire. In KORA F3 (2004/05), database supported computer software (IDOM: Instrument for databased assessment of medication) [37] was used. Daily amounts of regularly ingested nutrients per person were calculated for each survey from a database established by staff at the Helmholtz Zentrum München, which has been updated regularly [38]. Both total carotenoids and β-carotene intakes were investigated, but we restricted our main analyses to carotenoids, as the number of missing values was lower here.

Regular intake of each nutrient was investigated by dividing average intake amount per day into tertiles. Additionally, intake was analyzed binarily (regular vs no intake). For all analyses, the reference group comprised those who did not take the specific nutrient of interest. Cut-off points for tertiles were built from the means of the cut-off points from daily intake amounts at baseline and follow-up (see S1 Table).

#### Potential confounders

In both surveys, data regarding sex, age, physical activity, smoking status, alcohol intake, healthy diet, and medication intake were collected during a standardized face-to-face interview by trained medical staff [36]. For information on years of education and family history of diabetes, i.e., diabetes of the mother or father, only the baseline survey was considered. Participants who did not know whether their mother or father suffered or died from diabetes were added to the group that answered this question with “yes”. Education was defined through a combination of years of schooling and professional education and divided into a low (8–10 years) and high (11–17 years) level of education. Physical activity included frequency and duration of activity in summer and winter and was divided into four categories. Smoking status was divided into current (regular and irregular), former, and never smoking. Alcohol intake during the last week was assessed, and an average daily intake amount was calculated based on the alcohol intake during the last weekend and the last weekday prior to each examination. Sex-specific categories were created as follows: moderate intake (> 0 to < 20 g alcohol per day (g/d) (women) and > 0 to < 40 g/d (men)), high intake (≥ 20 g/d (women) and ≥ 40 g/d (men)), and no intake (0 g/d (men and women)). A food frequency questionnaire with 15 food items was used to build a healthy diet score according to the recommendations of the German Nutrition Society 1989 with higher values representing a healthier diet [39]. As food intake was only assessed through a short food frequency questionnaire in the total study sample which did not allow us to calculate intake levels for individual nutrients from diet, we restricted our analyses to intake of nutrients from dietary supplements. Medication that increased HbA_1c_ levels included beta-blockers, diuretics, and regular intake of systemic corticoids.

Anthropometric data (body mass index (BMI), waist–hip ratio (WHR)), information on hypertension, and cholesterol levels were collected during health examinations by trained medical staff. To calculate the BMI, participants’ weight and height were measured, and categories were applied according to the WHO. The WHR was calculated as the ratio between waist and hip circumferences and categorized as low (men < 1, women < 0.85) and high (men ≥ 1, women ≥ 0.85). Hypertension was defined as a systolic blood pressure ≥ 140 mmHg and/or diastolic blood pressure ≥ 90 mmHg or if the participant was taking antihypertensive medication and was aware of being hypertensive. Total and high density lipoprotein (HDL) cholesterol were measured using the CHOD-PAP method (Dade Behring) with CVs < 5% at baseline and follow-up.

### Statistical analyses

Age- and sex-adjusted mean levels of HbA_1c_ by categories of covariables and exposure variables were compared using analysis of covariance. To account for the intra-individual correlation, generalized estimating equation (GEE) models using the compound symmetry correlation structure were performed by the Statistical Analysis Systems (SAS) procedure GENMOD for investigation of the long-term association between vitamin/mineral intake from supplements/medications and HbA_1c_ levels. As results, the beta estimates of the interaction terms of each nutrient tertile of interest with time (coded 0/1) are presented (β_x_). These reflect the difference in change in HbA_1c_ levels between the nutrient tertile of interest and the reference group. Furthermore, beta estimates of time are displayed, which reflect the average change in HbA_1c_ levels in the reference group (β_t_). Estimates for each nutrient tertile of interest show the difference in HbA_1c_ levels at baseline compared with the reference group (β_0_).

Continuous covariates were investigated continuously if their association with the outcome was linear in cross-sectional analysis using univariable linear regression models; otherwise, they were categorized. The following variables were included in the models as potential confounders: sex, age (continuously), education (high/low), BMI (three groups), WHR (two groups), physical activity (four groups), smoking (three groups), alcohol intake (three sex-specific categories), healthy diet score (continuously), total/HDL cholesterol (continuously), actual hypertension (yes/no), diabetes of the father (yes/no), diabetes of the mother (yes/no), intake of HbA_1c_ increasing medication (yes/no).

The level of statistical significance was Bonferroni corrected and set at p < 0.0045 (11 investigated nutrients). Statistical analyses were performed using SAS version 9.3 (SAS Institute Inc, Cary, NC, USA).

### Sensitivity analyses

As sensitivity analyses, individuals were excluded who 1) developed diabetes during the 10-year follow-up period (n = 142); and 2) had baseline HbA_1c_ levels > 5.7% (n = 354) [40], as supplementation of nutrients in those with impaired glucose regulation might already be too late to exert beneficial effects [14]. Furthermore, stratified analyses according to smoking status were conducted to investigate the association between carotenoid intake and HbA_1c_ levels, because detrimental effects of β-carotene have been reported in smokers [41]. We also conducted longitudinal analyses only in those without any missing values in vitamin/mineral intake and HbA_1c_ values at baseline and follow-up (n = 2630).

## Results

### Descriptive analyses

Unadjusted mean (standard error of the mean, SEM) HbA_1c_ levels in the study population were 5.12 (0.01) at baseline (n = 4133) and 5.28 (0.01) at follow-up (n = 2774). Description of adjusted mean HbA_1c_ levels by participant characteristics is provided in Table 1. Adjusted mean levels of HbA_1c_ according to exposure categories are presented in Table 2. Regular intake of each investigated nutrient increased constantly from baseline over the 3-year to the 10-year follow-up (see S1 Fig). The proportion of individuals with constant regular intake of nutrients, i.e., regular intakes at baseline and follow-up, in relation to those with regular intake of that nutrient at baseline ranged between 10.0% (iron) and 31.0% (selenium).

**Table 1:** Characteristics of the study population with adjusted mean levels of HbA_1c_ at baseline (1994/95) and follow-up (2004/05).

		Baseline (1994/95)				Follow-up (2004/05)			
Characteristic		n Total (4447)	n HbA_1c_ (4133)	HbA_1c_ (SEM)*	p-value**	n Total (2774)	n HbA_1c_ (2766)	HbA_1c_ (SEM)*	p-value**
**Age (years at baseline)**					<0.001				<0.001
	25–34	893	833	4.97 (0.01)		592	589	5.13 (0.01)	
	35–44	949	892	5.04 (0.01)		674	671	5.22 (0.01)	
	45–54	904	867	5.13 (0.01)		649	649	5.31 (0.01)	
	55–64	910	840	5.20 (0.01)		558	557	5.37 (0.01)	
	65–74	791	701	5.31 (0.02)		301	300	5.49 (0.02)	
**Sex**					<0.001				0.290
	Male	2187	2093	5.04 (0.01)		1342	1338	5.28 (0.01)	
	Female	2260	2040	5.21 (0.01)		1432	1428	5.29 (0.01)	
**Education**					0.009				<0.001
	Low	2526	2339	5.14 (0.01)		1445	1442	5.32 (0.01)	
	High	1920	1794	5.10 (0.01)		1329	1324	5.25 (0.01)	
**BMI (kg/m** ^**2**^ **)**					<0.001				<0.001
	< 25	1611	1498	5.10 (0.01)		863	861	5.22 (0.01)	
	≥ 25 to < 30	1922	1817	5.12 (0.01)		1210	1207	5.28 (0.01)	
	≥ 30	866	793	5.18 (0.02)		680	679	5.37 (0.01)	
**Waist–hip ratio**					0.104				<0.001
	High	646	575	5.15 (0.02)		728	728	5.39 (0.01)	
	Low	3762	3538	5.12 (0.01)		2033	2027	5.25 (0.01)	
**Physical activity**					0.006				<0.001
	Very active	883	835	5.10 (0.01)		635	635	5.24 (0.01)	
	Moderately active	1074	1018	5.11 (0.01)		827	823	5.27 (0.01)	
	Less active	663	637	5.10 (0.02)		413	413	5.32 (0.02)	
	Inactive	1825	1642	5.15 (0.01)		891	889	5.31 (0.01)	
**Smoking**					0.571				0.087
	Current	1140	1067	5.14 (0.01)		527	526	5.31 (0.02)	
	Former	1348	1263	5.12 (0.01)		1015	1012	5.28 (0.01)	
	Never	1959	1803	5.12 (0.01)		1224	1222	5.27 (0.01)	
**Alcohol consumption**					<0.001				<0.001
	None	1440	1305	5.16 (0.01)		768	766	5.34 (0.01)	
	Moderate	2050	1929	5.13 (0.01)		1439	1436	5.28 (0.01)	
	High	956	898	5.07 (0.01)		557	556	5.22 (0.01)	
**Healthy diet**					0.786				0.356
	Unfavorable	1348	1258	5.13 (0.01)		682	680	5.27 (0.01)	
	Normal	904	848	5.12 (0.01)		424	423	5.26 (0.02)	
	Favorable	2190	2023	5.12 (0.01)		1358	1356	5.25 (0.01)	
**Hypertension**					0.679				<0.001
	Yes	1733	1598	5.12 (0.01)		1306	1303	5.31 (0.01)	
	No	2714	2535	5.13 (0.01)		1452	1449	5.26 (0.01)	
**Cholesterol/HDL ratio**					<0.001				<0.001
	< 3.5	1216	1158	5.08 (0.01)		1169	1169	5.24 (0.01)	
	≥ 3.5 to < 5.0	1631	1578	5.12 (0.01)		1061	1060	5.29 (0.01)	
	≥ 5.0	1456	1394	5.17 (0.01)		537	537	5.36 (0.01)	
**Diabetes mellitus (father)**					0.001				0.010
	Yes	1136	1054	5.16 (0.01)		696	694	5.31 (0.01)	
	No	3309	3077	5.11 (0.01)		2077	2071	5.27 (0.01)	
**Diabetes mellitus (mother)**					<0.001				0.007
	Yes	937	874	5.17 (0.01)		544	542	5.32 (0.01)	
	No	3508	3257	5.11 (0.01)		2229	2223	5.27 (0.01)	
**HbA**_**1c**_ **↑ medication**					0.736				<0.001
	Yes	555	499	5.13 (0.02)		677	675	5.34 (0.01)	
	No	3888	3630	5.12 (0.01)		2090	2086	5.26 (0.01)	

BMI = body mass index, HbA_1c_ = hemoglobin A_1c_, HDL = high density lipoprotein, SEM = standard error of the mean

*age- and sex-adjusted mean HbA_1c_, for adjustment age was treated continuously, age was only sex adjusted, sex was only age adjusted

**p for adjusted mean difference (analysis of covariance); numbers of people in categories in column “n Total” do not sum up to the number in the total study population because of missing values.

**Table 2:** Adjusted mean levels of HbA_1c_ by categories of exposure variables at baseline (1994/95) and follow-up (2004/05).

		Baseline (1994/95)				Follow-up (2004/05)			
Nutrient	Category	n Total (4447)	n HbA_1c_ (4133)	HbA_1c_ (SEM)*	p-value**	n Total (2774)	n HbA_1c_ (2766)	HbA_1c_ (SEM)*	p-value**
**Vitamin E**					0.256				0.934
	No intake	4037	3744	5.13 (0.01)		2321	2316	5.28 (0.01)	
	Tertile 1	104	97	5.18 (0.04)		126	126	5.28 (0.03)	
	Tertile 2	84	81	5.11 (0.05)		132	132	5.26 (0.03)	
	Tertile 3	88	80	5.04 (0.05)		148	147	5.27 (0.03)	
**Vitamin C**					0.456				0.410
	No intake	3882	3597	5.13 (0.01)		2255	2249	5.29 (0.01)	
	Tertile 1	119	112	5.13 (0.04)		163	163	5.27 (0.03)	
	Tertile 2	80	75	5.14 (0.05)		107	107	5.23 (0.03)	
	Tertile 3	114	108	5.06 (0.04)		136	136	5.29 (0.03)	
**Vitamin D**					0.764				0.049
	No intake	4328	4026	5.13 (0.01)		2500	2494	5.29 (0.01)	
	Tertile 1	30	26	5.06 (0.08)		45	45	5.17 (0.05)	
	Tertile 2	42	38	5.09 (0.07)		148	148	5.30 (0.03)	
	Tertile 3	15	14	5.05 (0.11)		50	50	5.24 (0.05)	
**Vitamin B**_**1**_					0.873				0.491
	No intake	4101	3810	5.13 (0.01)		2453	2447	5.28 (0.01)	
	Tertile 1	83	79	5.16 (0.05)		122	122	5.28 (0.03)	
	Tertile 2	53	49	5.09 (0.06)		62	62	5.23 (0.04)	
	Tertile 3	88	78	5.09 (0.05)		90	90	5.31 (0.04)	
**Folic acid**					0.291				0.745
	No intake	4204	3901	5.13 (0.01)		2437	2431	5.28 (0.01)	
	Tertile 1	53	50	5.10 (0.06)		62	62	5.32 (0.04)	
	Tertile 2	55	55	5.13 (0.06)		126	126	5.27 (0.03)	
	Tertile 3	37	33	4.97 (0.07)		106	106	5.29 (0.03)	
**Carotenoids**					0.199				0.420
	No intake	4375	4066	5.12 (0.01)		2608	2602	5.28 (0.01)	
	Tertile 1	18	16	5.02 (0.11)		40	40	5.31 (0.05)	
	Tertile 2	13	13	5.11 (0.12)		58	58	5.28 (0.04)	
	Tertile 3	18	17	5.33 (0.10)		46	46	5.20 (0.05)	
**Calcium**					0.778				0.678
	No intake	4073	3789	5.12 (0.01)		2398	2392	5.28 (0.01)	
	Tertile 1	106	98	5.10 (0.04)		69	69	5.25 (0.04)	
	Tertile 2	93	87	5.14 (0.05)		121	121	5.27 (0.03)	
	Tertile 3	63	56	5.12 (0.06)		131	131	5.28 (0.03)	
**Magnesium**					0.652				0.118
	No intake	3963	3681	5.13 (0.01)		2253	2247	5.29 (0.01)	
	Tertile 1	133	124	5.14 (0.04)		122	122	5.26 (0.03)	
	Tertile 2	132	121	5.07 (0.04)		155	155	5.24 (0.03)	
	Tertile 3	75	72	5.12 (0.05)		144	144	5.32 (0.03)	
**Zinc**					0.329				0.794
	No intake	4365	4060	5.12 (0.01)		2480	2475	5.28 (0.01)	
	Tertile 1	46	40	5.09 (0.07)		41	41	5.27 (0.05)	
	Tertile 2	1	1	4.49 (0.42)		10	10	5.26 (0.11)	
	Tertile 3	8	8	5.30 (0.15)		202	201	5.27 (0.02)	
**Iron**					0.886				0.345
	No intake	4303	4005	5.13 (0.01)		2605	2599	5.28 (0.01)	
	Tertile 1	32	29	5.09 (0.08)		103	103	5.27 (0.03)	
	Tertile 2	19	17	5.07 (0.10)		16	16	5.17 (0.08)	
	Tertile 3	59	51	5.09 (0.06)		23	23	5.24 (0.07)	
**Selenium**					0.845				0.201
	No intake	4404	4093	5.13 (0.01)		2569	2563	5.29 (0.01)	
	Tertile 1	5	5	5.01 (0.19)		112	112	5.25 (0.03)	
	Tertile 2	13	12	5.10 (0.12)		27	27	5.27 (0.07)	
	Tertile 3	11	11	5.09 (0.13)		37	37	5.29 (0.06)	

HbA_1c_ = hemoglobin A_1c_, SEM = standard error of the mean

*age- and sex-adjusted mean HbA_1c_, for adjustment age was treated continuously

**p for adjusted mean difference (analysis of covariance); numbers of people in categories in column “n Total” do not sum up to the number in the total study population because of missing values and because subjects with intakes as needed are not listed.

### Main analysis

After 10 years, the HbA_1c_ levels of participants who comprised the reference group (no intake) for each investigated nutrient increased by about 0.1% from baseline (see β_t_ in Table 3). Participants with carotenoid intakes in the upper tertile had a 0.26% lower increase in HbA_1c_ values after 10 years than those with no intake of carotenoids (see β_x_ in **Table 3**). It is noteworthy that participants with carotenoid intakes in the upper tertile had 0.22% higher HbA_1c_ levels at baseline compared with the reference group (see β_0_ in Table 3), even though this difference was marginally not statistically significant after Bonferroni correction (p = 0.005). The intake of none of the other investigated nutrient tertiles was significantly associated with concentrations of HbA_1c_ after correction for multiple testing (see **Table 3**). Treating the exposure to regular intake of each nutrient as binary variables (regular intake vs no intake) yielded analogous findings, whereas the association of regular carotenoid intake with HbA_1c_ levels was not statistically significant (data not shown).

**Table 3:** Longitudinal association between regular supplement intake and HbA_1c_ (1994/95–2004/05); results from generalized estimating equations (GEE).

			Tertile 1		Tertile 2		Tertile 3	
	Model	ß_t_ (95% CI)	ß_0_ (95% CI)	ß_x_ (95% CI)	ß_0_ (95% CI)	ß_x_ (95% CI)	ß_0_ (95% CI)	ß_x_ (95% CI)
**Vitamin E**								
	Sex- and age-adjusted	**0.09 (0.07 to 0.11)**	0.07 (-0.02 to 0.15)	-0.08 (-0.18 to 0.02)	-0.00 (-0.09 to 0.09)	-0.02 (-0.12 to 0.08)	-0.09 (-0.18 to -0.01)	0.07 (-0.02 to 0.17)
	Fully adjusted*	**0.10 (0.08 to 0.12)**	0.08 (-0.00 to 0.17)	-0.10 (-0.20 to 0.01)	0.01 (-0.08 to 0.10)	-0.01 (-0.11 to 0.09)	-0.08 (-0.16 to 0.01)	0.08 (-0.02 to 0.17)
**Vitamin C**								
	Sex- and age-adjusted	**0.10 (0.08 to 0.11)**	0.02 (-0.05 to 0.10)	-0.05 (-0.13 to 0.04)	0.01 (-0.09 to 0.11)	-0.09 (-0.20 to 0.03)	-0.06 (-0.13 to 0.01)	0.06 (-0.03 to 0.14)
	Fully adjusted*	**0.11 (0.09 to 0.13)**	0.04 (-0.04 to 0.11)	-0.05 (-0.13 to 0.04)	0.03 (-0.08 to 0.13)	-0.09 (-0.21 to 0.02)	-0.05 (-0.12 to 0.03)	0.05 (-0.04 to 0.14)
**Vitamin D**								
	Sex- and age-adjusted	**0.10 (0.08 to 0.11)**	-0.08 (-0.23 to 0.06)	-0.03 (-0.19 to 0.13)	-0.02 (-0.15 to 0.10)	-0.00 (-0.14 to 0.14)	-0.04 (-0.20 to 0.12)	-0.04 (-0.21 to 0.13)
	Fully adjusted*	**0.11 (0.09 to 0.13)**	-0.05 (-0.20 to 0.10)	-0.04 (-0.20 to 0.12)	-0.01 (-0.14 to 0.12)	-0.03 (-0.17 to 0.10)	-0.06 (-0.23 to 0.12)	-0.00 (-0.20 to 0.19)
**Vitamin B**_**1**_								
	Sex- and age-adjusted	**0.09 (0.07 to 0.11)**	0.05 (-0.04 to 0.14)	-0.08 (-0.19 to 0.02)	-0.03 (-0.14 to 0.09)	-0.02 (-0.15 to 0.11)	-0.04 (-0.13 to 0.06)	0.05 (-0.06 to 0.17)
	Fully adjusted*	**0.10 (0.09 to 0.12)**	0.06 (-0.03 to 0.15)	-0.07 (-0.18 to 0.04)	-0.01 (-0.12 to 0.10)	-0.00 (-0.13 to 0.13)	-0.02 (-0.12 to 0.07)	0.01 (-0.10 to 0.12)
**Folic acid**								
	Sex- and age-adjusted	**0.09 (0.07 to 0.11)**	0.00 (-0.10 to 0.11)	0.02 (-0.11 to 0.16)	0.02 (-0.09 to 0.13)	-0.06 (-0.18 to 0.07)	-0.16 (-0.30 to -0.02)	0.13 (-0.01 to 0.28)
	Fully adjusted*	**0.10 (0.08 to 0.12)**	0.01 (-0.09 to 0.12)	0.02 (-0.11 to 0.15)	0.02 (-0.09 to 0.14)	-0.04 (-0.17 to 0.09)	-0.13 (-0.26 to -0.01)	0.12 (-0.02 to 0.25)
**Carotenoids**								
	Sex- and age-adjusted	**0.09 (0.07 to 0.11)**	-0.08 (-0.30 to 0.13)	0.08 (-0.16 to 0.32)	-0.00 (-0.27 to 0.26)	-0.00 (-0.28 to 0.27)	0.19 (0.04 to 0.35)	**-0.27 (-0.45 to -0.10)**
	Fully adjusted*	**0.10 (0.09 to 0.12)**	-0.08 (-0.30 to 0.13)	0.07 (-0.17 to 0.31)	0.02 (-0.25 to 0.30)	-0.03 (-0.31 to 0.25)	0.22 (0.07 to 0.38)	**-0.26 (-0.43 to -0.08)**
**Calcium**								
	Sex- and age-adjusted	**0.10 (0.08 to 0.12)**	-0.02 (-0.10 to 0.07)	-0.04 (-0.14 to 0.07)	0.04 (-0.04 to 0.12)	-0.07 (-0.17 to 0.02)	-0.00 (-0.11 to 0.11)	-0.04 (-0.16 to 0.08)
	Fully adjusted*	**0.11 (0.09 to 0.13)**	0.00 (-0.08 to 0.09)	-0.08 (-0.18 to 0.02)	0.03 (-0.05 to 0.11)	-0.05 (-0.14 to 0.05)	-0.01 (-0.12 to 0.10)	-0.04 (-0.16 to 0.08)
**Magnesium**								
	Sex- and age-adjusted	**0.09 (0.08 to 0.11)**	0.02 (-0.06 to 0.09)	-0.04 (-0.13 to 0.04)	-0.06 (-0.13 to 0.01)	0.00 (-0.08 to 0.08)	0.00 (-0.10 to 0.11)	0.00 (-0.12 to 0.12)
	Fully adjusted*	**0.11 (0.09 to 0.13)**	0.03 (-0.05 to 0.10)	-0.06 (-0.15 to 0.03)	-0.05 (-0.12 to 0.02)	0.00 (-0.08 to 0.08)	-0.00 (-0.10 to 0.10)	0.02 (-0.10 to 0.14)
**Iron**								
	Sex- and age-adjusted	**0.09 (0.08 to 0.11)**	-0.06 (-0.19 to 0.06)	0.04 (-0.09 to 0.18)	-0.06 (-0.24 to 0.13)	-0.08 (-0.27 to 0.11)	-0.02 (-0.13 to 0.09)	-0.06 (-0.23 to 0.11)
	Fully adjusted*	**0.11 (0.09 to 0.12)**	-0.03 (-0.15 to 0.09)	0.00 (-0.13 to 0.14)	-0.05 (-0.24 to 0.14)	-0.06 (-0.26 to 0.14)	-0.00 (-0.11 to 0.11)	-0.10 (-0.26 to 0.06)
**Selenium**								
	Sex- and age-adjusted	**0.09 (0.07 to 0.11)**	-0.05 (-0.25 to 0.15)	0.02 (-0.19 to 0.23)	-0.00 (-0.29 to 0.29)	-0.02 (-0.33 to 0.30)	-0.07 (-0.34 to 0.19)	0.09 (-0.18 to 0.37)
	Fully adjusted*	**0.10 (0.09 to 0.12)**	-0.01 (-0.17 to 0.16)	-0.03 (-0.20 to 0.15)	0.01 (-0.29 to 0.31)	-0.06 (-0.36 to 0.25)	-0.05 (-0.33 to 0.24)	0.09 (-0.21 to 0.39)

CI = confidence interval, HbA_1c_ = hemoglobin A_1c_; ß_t_ = average change in HbA_1c_ between baseline (1994/95) and follow-up (2004/05) in the reference group “no intake”, ß_0_ = difference in HbA_1c_ at baseline compared with the reference group “no intake”, ß_x_ = difference in change in HbA_1c_ compared with the reference group “no intake”; significant associations after Bonferroni correction are presented in bold font

*adjusted for: sex, age, BMI, waist–hip ratio, physical activity, smoking, alcohol intake, healthy diet, total/HDL cholesterol, hypertension, diabetes of the father, diabetes of the mother, intake of HbA_1c_ increasing medication; as building tertiles with nearly similar numbers of people was not possible for zinc intake, results for zinc intake are not presented.

### Sensitivity analyses

Excluding 142 participants who developed diabetes during the 10-year follow-up resulted in similar findings to the main analysis, apart from the fact that the inverse association between the upper carotenoid tertile and change in HbA_1c_ levels was not statistically significant (data not shown). Like in the main analysis, after exclusion of 354 participants with baseline HbA_1c_ levels > 5.7%, the increase in HbA_1c_ levels in individuals with intakes of carotenoids in the upper tertile was significantly lower (β_x_ = –0.29% (95% CI: –0.45 to –0.12)) compared with individuals with no intake of carotenoids (p = 0.0006). Unlike in the main analysis, the difference in baseline HbA_1c_ levels of 0.25% (95% CI: 0.11 to 0.39) between those with the highest intakes of carotenoids compared with those with no intake was statistically significant.

**Fig 1:**
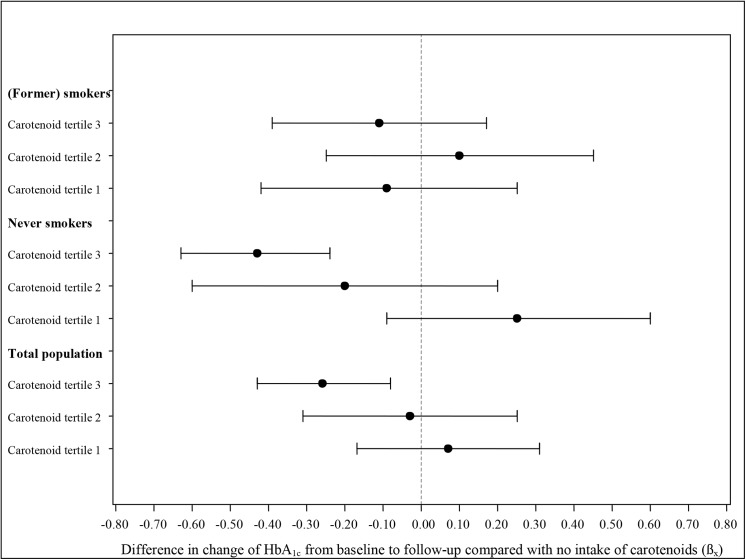
Longitudinal association between regular carotenoid intake amount from supplements and medications in tertiles and levels of HbA_1c_ over 10 years (1994/95 to 2004/05). n_Total population_ = 4447, n_never smokers_ = 1959, n_(former) smokers_ = 2498; 10 people who stated they were never smokers at baseline but smokers at follow-up were partly used for both strata for the appropriate time point; HbA_1c_ beta estimates (β_x_) result from fully adjusted generalized estimating equation models and refer to interaction terms with time (represents the difference in change in HbA_1c_ levels compared with those with no intake of carotenoids, see β_x_ in Table 3 for total population); cut-off point for tertiles 1 and 2: 1.9 mg/d; for tertiles 2 and 3: 6.8 mg/d.

In the smoking status-stratified analyses of carotenoid intake and HbA_1c_ levels, the results for current and former smokers were pooled together, as they did not differ substantially (data not shown). A longitudinal inverse association of the upper carotenoid tertile with HbA_1c_ level was significant in participants who never smoked (β_x_ = –0.43% (95% CI: –0.63 to –0.24), p < 0.0001), but not in current or former smokers (β_x_ = –0.11% (95% CI: –0.39 to 0.17), p = 0.451) (see Fig 1). Never smokers with carotenoid intakes in the upper tertile had higher baseline HbA_1c_ values than never smokers with no intake of carotenoids (β_0_ = 0.33 (95% CI: 0.18 to 0.48), p < 0.0001)_._

The results when restricting the study population to participants without any missing values in exposure and outcome at baseline and follow-up were similar to the main analysis (data not shown).

## Discussion

We investigated the longitudinal association of regular intake of vitamin E, vitamin C, vitamin D, vitamin B_1_, folic acid, carotenoids, calcium, magnesium, zinc, iron, and selenium from supplements and medications with levels of HbA_1c_ in the general non-diabetic population. The results indicate that there is an inverse association of regular carotenoid intakes above 6.8 mg/d with concentrations of HbA_1c_ after 10 years and no significant association of intake of the other investigated vitamins and minerals with HbA_1c_ levels. The observed reduction of 0.3 absolute percentage points in HbA_1c_ levels in the upper carotenoid tertile can be regarded as clinically relevant, as a reduction of 0.1% in the general non-diabetic population could lead to a reduction in mortality of up to 6% over a period of 6 years [9].

### Comparison with the literature

Previous intervention studies examining the effect of use of vitamins/minerals on HbA_1c_ levels have mostly been conducted in diabetic or prediabetic subjects and found inconsistent results. Significant inverse associations with HbA_1c_ levels were reported for vitamins E, C, D, B_1_, folic acid, calcium, magnesium, zinc, iron, and selenium supplemented either alone or in combination [12,19,21,22,24,26,42]. Other intervention studies found no significant associations for vitamins E, C, D, B_1_, folic acid, carotenoids, calcium, magnesium, and zinc [10,11,13–15,17,18,20,23,25]. In contrast, significant positive associations were also observed for vitamin D, folic acid, and selenium [16,27,43].

Inadequacy of baseline nutrient status might influence the efficacy of supplementation, as single studies that reported a protective role for supplemented nutrients (iron, vitamin D with calcium) were conducted in those with inadequate baseline status [21,26]. Furthermore, a recently published meta-analysis on vitamin E supplementation found that HbA_1c_ lowering effects might rather be present in people with low baseline vitamin E status [10]. The same study reported that vitamin E dosages above 400 mg/d might be necessary to result in decreased HbA_1c_ levels. In the present study, two thirds of all subjects who regularly supplemented vitamin E ingested daily amounts of less than 66 mg.

One explanation for the null findings in some of the above mentioned studies is that all were conducted in (pre-)diabetic or high risk populations. The state of dysglycemia might already be too advanced to facilitate beneficial effects of vitamin supplementation [11,14]. Therefore, further intervention studies are warranted in non-diabetic and also in non-prediabetic individuals to investigate the relationship between vitamin/mineral intake and HbA_1c_ levels.

### Carotenoids

In the present study, regular intake of > 6.8 mg of carotenoids per day from supplements and medications, which corresponds to the upper tertile, was associated with an about 0.3% lower increase in HbA_1c_ concentrations after 10 years. Similar dose–effect results were presented by Akbaraly et al. from the Epidemiology of Vascular Ageing (EVA) study; however, they investigated plasma carotenoid levels as exposure with regard to other diabetes related outcomes in elderly volunteers [44]. They found that the risk of dysglycemia was significantly lower in the highest plasma carotenoid quartile after 9 years of follow-up.

Prospective studies on the association of carotenoids (intake or plasma/serum levels) with HbA_1c_ levels as an outcome have not, to our knowledge, been published so far. Cross-sectional studies in healthy subjects reported an inverse association [31,32], or no association [30]. A randomized controlled trial (RCT) detected no significant effect with regard to lowering of HbA_1c_ levels [13]. However, the trial was conducted in people with type 2 diabetes [13]. To our knowledge, there is currently no RCT that investigated the effect of carotenoids on HbA_1c_ levels in healthy individuals. Also, the safety of carotenoid supplementation in higher dosages has to be confirmed in future studies, as detrimental effects have been found for β-carotene not only in smokers with dosages above 20 mg/d [41], but also in a pooled analysis of diverse populations with dosages ranging from 15 to 50 mg/d [45]. The main carotenoid ingested in the present study population was β-carotene. Some 98.0% and 68.1%, respectively, of all those who regularly ingested carotenoids at baseline and follow-up took β-carotene. Mean (SD) intake amount in the upper tertile of carotenoids was 15.4 (9.2) mg with a maximum value of 50 mg.

#### Mechanism

A potential mechanism for a HbA_1c_ lowering effect of carotenoids might be their antioxidant activity [46], which is closely related to protein glycosylation [47]. However, controlling for several antioxidative markers in the EVA study mentioned above did not change the result of a lower risk of dysglycemia with higher plasma carotenoids [44]. Furthermore, intake of the other investigated antioxidants (vitamin E, vitamin C, zinc, and selenium) from supplements or medications was not significantly associated with lower HbA_1c_ values in the present investigation. Recently, Bumke-Vogt et al. [48] reported that, apart from carotenoids, another subgroup of phytochemicals resulted in down-regulation of gluconeogenic gene expression in human hepatoma cells, an effect not attributed to their antioxidant activity [48]. This suggests that phytochemicals might exert other effects than hitherto known.

#### Smoking status

Stratified analyses according to smoking status showed that an inverse association of carotenoids and HbA_1c_ levels is only present in those who have never smoked. These results support previous findings by Hozawa et al., who investigated serum carotenoid levels as exposure and diabetes incidence and insulin concentrations as outcomes in the Coronary Artery Risk Development in Young Adults (CARDIA) study [49]. The authors concluded that higher serum carotenoid levels are significantly associated with a lower risk of diabetes and insulin resistance after 15 years only in non-smokers, but not in smokers.

### Strengths and limitations

A limitation of the present study is that measurements of HbA_1c_ and covariables were only available for two time points. Intake of supplements was assessed at three time points. Furthermore, there were no detailed data available on nutrient intakes from diet, and we only adjusted for a diet score that represents a healthy or an unhealthy eating pattern. However, we believe that dietary habits remained rather constant during the study period, as the categorized healthy diet score remained the same in 56% of the participants with available information at baseline and follow-up. An important limitation is the small (and also unequal) number of participants in some nutrient tertiles, which might have underpowered the present analyses. Furthermore, variation in baseline HbA_1c_ levels was high in specific tertile groups, and the results might be driven by outliers. Moreover, plasma levels were not measured, although these do not always represent intake of nutrients, especially if a high turnover is present, such as with antioxidant levels in smokers [50]. Inadequacy of baseline status of nutrients was not considered in the present analysis, but might influence the efficacy of supplementation [10,21,26]. With respect to vitamin E, ingested dosages might have been too low. Another important point to consider is that participants with the highest intakes of carotenoids at baseline had relatively high baseline levels of HbA_1c_. Therefore, regression to the mean might be present and might lead to biased results with regard to the inverse longitudinal association. We also cannot exclude the possibility that the results are due to residual confounding, as this is an observational study, even if the beta estimates did not differ greatly between the raw and fully adjusted models.

The strengths of the present study include the long follow-up duration of 10 years, the availability of important covariables not only at baseline but also at follow-up, and the thorough assessment of vitamin/mineral intake from supplements and medications. The availability of average daily intake amounts of nutrients allowed the conduct of dose–effect analyses, which are crucial in the investigation of physiological nutrient effects. Furthermore, this represents a population-based study of healthy non-diabetic individuals. Therefore, the results can be transferred to the general population. Finally, HbA_1c_ exhibits a lower biological variability compared with other glycemic outcomes [51] and is strongly associated with cardiovascular disease risk in non-diabetic people [8].

### Future perspectives

Larger prospective studies on vitamin/mineral intake or blood levels and HbA_1c_ concentrations are needed to confirm the results of the present analyses. If the results regarding a significant inverse association of higher carotenoid intakes with HbA_1c_ levels are replicated, RCTs can be conducted, especially in non-smokers and those without diabetes, to investigate the causal effect of carotenoid dosages > 6.8 mg/d in lowering HbA_1c_ concentrations. If these studies confirm the present findings, supplementation of carotenoids might be a useful strategy in preventing the development of type 2 diabetes as well as cardiovascular complications and mortality in the general or non-smoking population.

## Supporting Information

S1 Fig Regular intake of dietary supplements over 10 years. Data are derived from the MONICA (Monitoring of Trends and Determinants in Cardiovascular Diseases) S3 survey in 1994/95 (n = 4447), from a postal questionnaire in 1997/98 (n = 2998), and from the KORA (Cooperative Health Research in the Region of Augsburg) F3 survey in 2004/05 (n = 2774); all surveys longitudinally investigated the same individuals.
(TIF)

S1 Table Cut-off points of daily intake amounts for building tertiles in baseline and follow-up surveys and their mean values. For building of tertiles, cut-off values were included in the lower category.
(DOC)
