# Supplementary figures and images for: Intake of Vitamin and Mineral Supplements and Longitudinal Association with HbA1c Levels in the General Non-Diabetic Population—Results from the MONICA/KORA S3/F3 Study
Source: PLoS One. 2015 Oct 16;10(10):e0139244. doi: 10.1371/journal.pone.0139244 (PMC4608810; doi:10.1371/journal.pone.0139244)

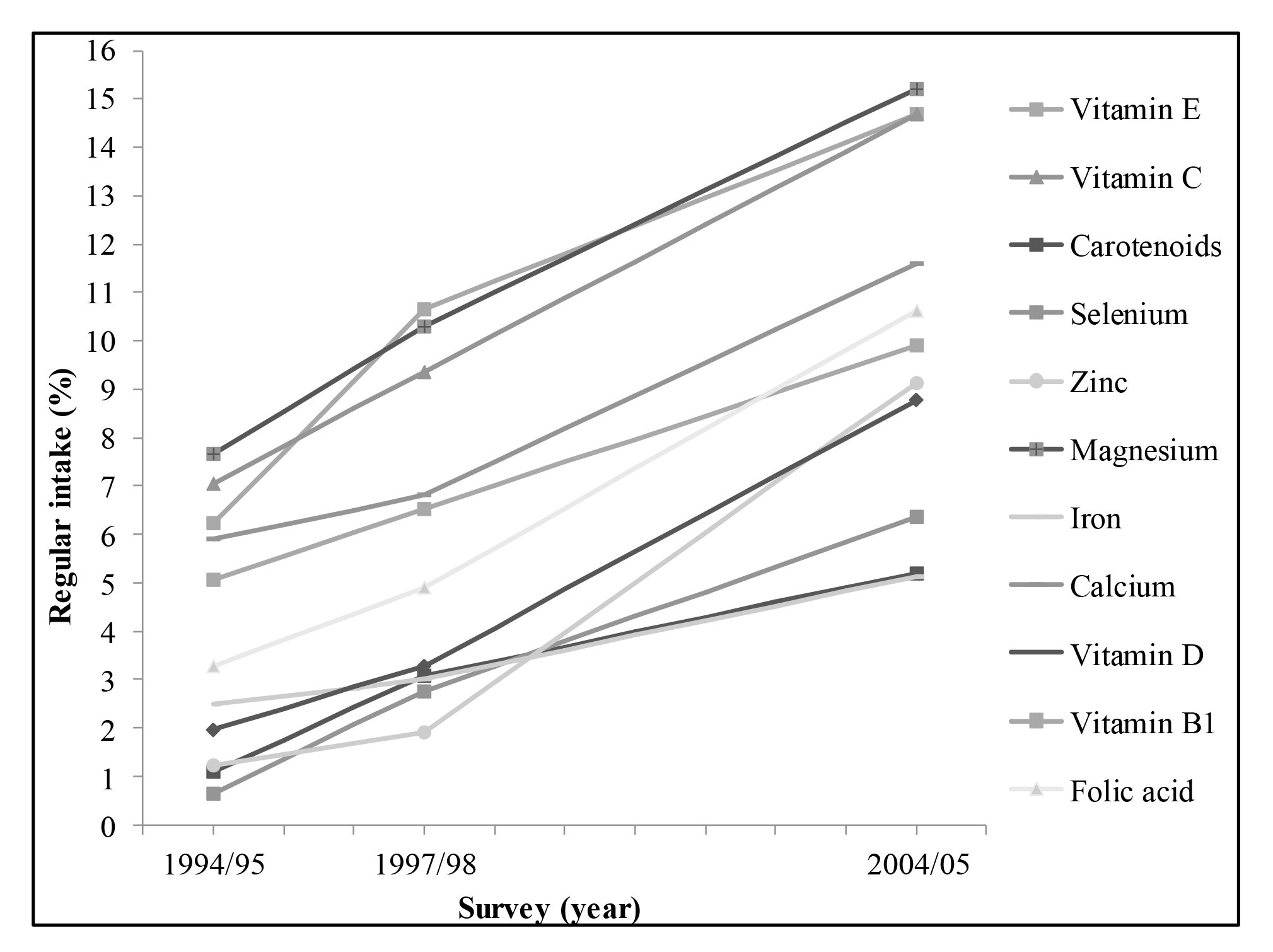

Supplement: S1 Fig — Data are derived from the MONICA (Monitoring of Trends and Determinants in Cardiovascular Diseases) S3 survey in 1994/95 (n = 4447), from a postal questionnaire in 1997/98 (n = 2998), and from the KORA (Cooperative Health Research in the Region of Augsburg) F3 survey in 2004/05 (n = 2774); all surveys longitudinally investigated the same individuals. (TIF) [file pone.0139244.s001.tif]
