# Supplementary material for: Intake of Vitamin and Mineral Supplements and Longitudinal Association with HbA1c Levels in the General Non-Diabetic Population—Results from the MONICA/KORA S3/F3 Study
Source: PLoS One. 2015 Oct 16;10(10):e0139244. doi: 10.1371/journal.pone.0139244 (PMC4608810; doi:10.1371/journal.pone.0139244)
Supplement: S1 Table — For building of tertiles, cut-off values were included in the lower category. (DOC) [file pone.0139244.s002.doc]

**Supporting Information**

**Intake of vitamin and mineral supplements and longitudinal association with HbA_1c_ levels in the general non-diabetic population —Results from the MONICA/KORA S3/F3 study**

S. Schwab ^1^, A. Zierer ^1^, M. Heier ^1,4^, B. Fischer ^2^, C. Huth ^1,3^, J. Baumert ^1,3^, C. Meisinger ^1,4^, A. Peters ^1,3^, B. Thorand ^1,3*^

^1^ Institute of Epidemiology II, Helmholtz Zentrum München, German Research Center for Environmental Health, Neuherberg, Germany

^2^ Department of Epidemiology and Preventive Medicine, University of Regensburg, Regensburg, Germany

^3^ German Center for Diabetes Research (DZD e.V.), Neuherberg, Germany

^4^ MONICA/KORA Myocardial Infarction Registry, Central Hospital of Augsburg, Augsburg, Germany

****Corresponding author***

E-mail: thorand@helmholtz-muenchen.de (BT)

**S1 Fig. Regular intake of dietary supplements over 10 years.** Data are derived from the MONICA (Monitoring of Trends and Determinants in Cardiovascular Diseases) S3 survey in 1994/95 (n=4447), from a postal questionnaire in 1997/98 (n=2998), and from the KORA (Cooperative Health Research in the Region of Augsburg) F3 survey in 2004/05 (n=2774); all surveys longitudinally investigated the same individuals.


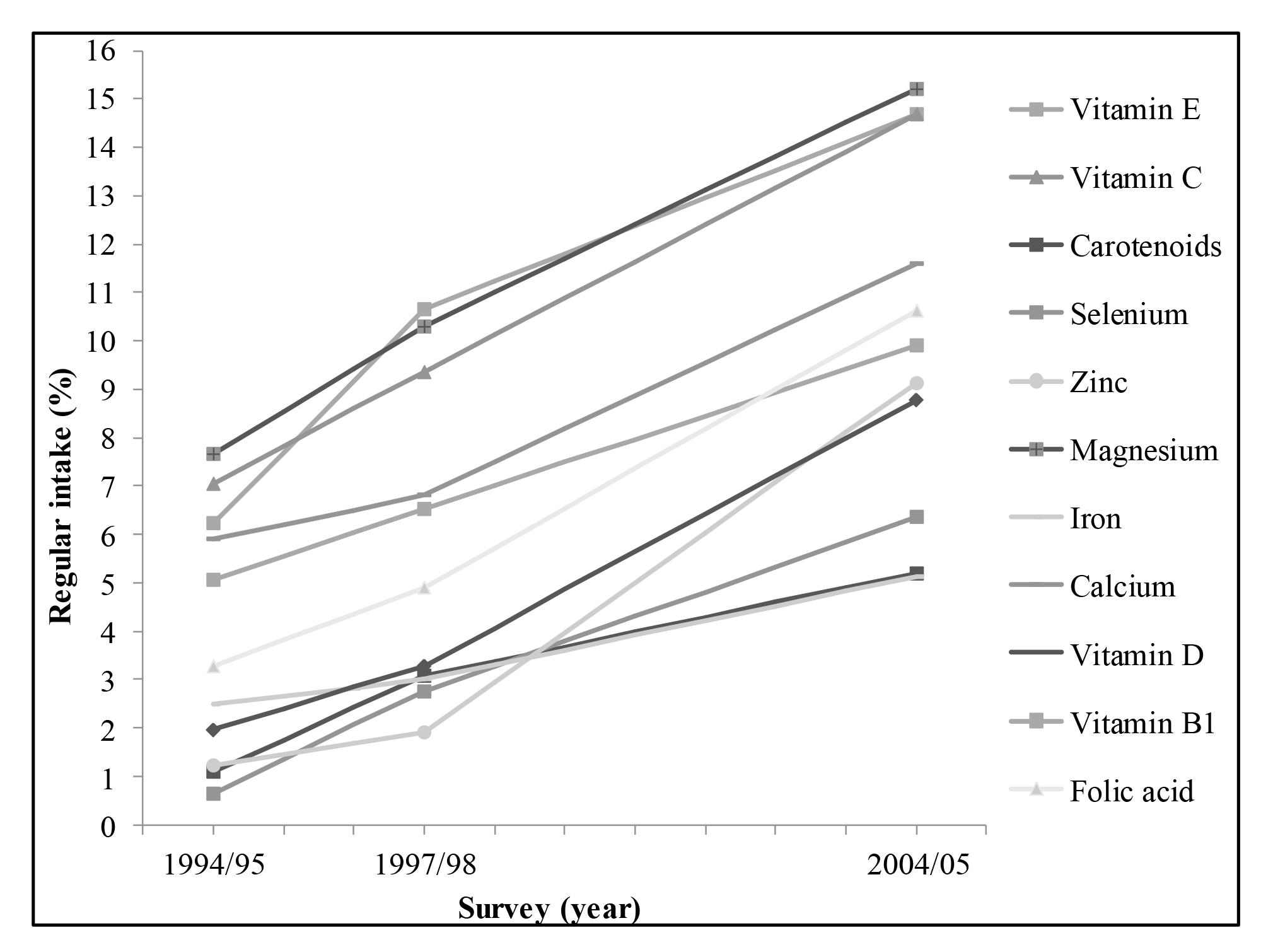


S1 Table. Cut-off points of daily intake amounts for building tertiles in baseline and follow-up survey and their mean values.

|  |  | **Baseline (1994/95)** | |  | **Follow-up (2004/05)** | |  | **Mean baseline and follow-up** | |
| --- | --- | --- | --- | --- | --- | --- | --- | --- | --- |
| **Nutrient** | **Unit** | **Tertile 1/2** | **Tertile 2/3** |  | **Tertile 1/2** | **Tertile 2/3** |  | **Tertile 1/2** | **Tertile 2/3** |
| **Vitamin E** | mg/d | 10.0 | 50.0 |  | 12.0 | 81.2 |  | 11.0 | 65.6 |
| **Vitamin C** | mg/d | 75.0 | 240.0 |  | 75.0 | 202.5 |  | 75.0 | 221.3 |
| **Vitamin D** | µg/d | 3.6 | 10.0 |  | 5.0 | 10.0 |  | 4.3 | 10.0 |
| **Vitamin B_1_** | mg/d | 1.4 | 3.0 |  | 1.4 | 2.4 |  | 1.4 | 2.7 |
| **Folic acid** | µg/d | 160.0 | 272.0 |  | 200.0 | 400.0 |  | 180.0 | 336.0 |
| **Carotenoids** | mg/d | 1.8 | 7.5 |  | 2.0 | 6.0 |  | 1.9 | 6.8 |
| **Calcium** | mg/d | 94.0 | 400.0 |  | 162.0 | 500.0 |  | 128.0 | 450.0 |
| **Magnesium** | mg/d | 48.6 | 150.0 |  | 75.0 | 156.6 |  | 61.8 | 153.3 |
| **Zinc** | mg/d | 0.3 | 1.0 |  | 5.0 | 6.3 |  | 2.7 | 3.7 |
| **Iron** | mg/d | 10.0 | 18.3 |  | 4.0 | 5.1 |  | 7.0 | 11.7 |
| **Selenium** | µg/d | 50.0 | 75.0 |  | 25.0 | 50.0 |  | 37.5 | 62.5 |

For building of tertiles, cut-off values were included in the lower category.
